# The cognitive profile of Friedreich ataxia: a systematic review and meta-analysis

**DOI:** 10.1186/s12883-022-02615-3

**Published:** 2022-03-17

**Authors:** Gilles Naeije, Jörg B Schulz, Louise A Corben

**Affiliations:** 1 Laboratoire de Cartographie fonctionnelle du Cerveau (LCFC), UNI – ULB Neuroscience Institute, Université libre de Bruxelles (ULB), 808 Lennik Street, 1070 Brussels, Belgium; 2 Department of Neurology, RWTH Aachen University Hospital, Pauwelsstraße 30, Aachen, Germany; 3 JARA Institute Molecular Neuroscience and Neuroimaging, Forschungszentrum Jülich GmbH and RWTH Aachen University, 52074 Aachen, Germany; 4 Bruce Lefroy Centre for Genetic Health Research, Murdoch Children’s Research Institute, Parkville, Australia; 5 Department of Paediatrics, The University of Melbourne, Parkville, Australia; 6 Turner Institute for Brain and Mental Health, Monash University, Clayton, Australia

**Keywords:** Cognition, Cerebellum, Friedreich ataxia, Cerebellar cognitive affective syndrome

## Abstract

**Background:**

Study the cognitive profile of individuals with Friedreich ataxia (FRDA) and seek evidence for correlations between clinical, genetic and imaging characteristics and neuropsychological impairments.

**Methods:**

Based on PRISMA guidelines, a meta-analysis was realized using the Pubmed and Scopus databases to identify studies (1950–2021) reporting neuropsychological test results in genetically confirmed FRDA and control participants in at least one of the following cognitive domains: attention/executive, language, memory and visuo-spatial functions as well as emotion. Studies using identical outcomes in a minimum of two studies were pooled. Pooled effect sizes were calculated with Cohen’s *d*.

**Results:**

Eighteen studies were included. Individuals with FRDA displayed significantly lower performance than individuals without FRDA in most language, attention, executive function, memory visuospatial function, emotion regulation and social cognitive tasks. Among the included studies, thirteen studies examined the relationship between neuropsychological test results and clinical parameters and reported significant association with disease severity and six studies reviewed the relationship between neuroimaging measures and cognitive performance and mainly reported links between reduced cognitive performance and changes in cerebellar structure.

**Conclusions:**

Individuals with FRDA display significantly lower performances in many cognitive domains compared to control participants. The spectrum of the cognitive profile alterations in FRDA and its correlation with disease severity and cerebellar structural parameters suggest a cerebellar role in the pathophysiology of FRDA cognitive impairments.

## Background

Friedreich ataxia (FRDA) is the most common autosomal recessive ataxia [1]. Most patients are homozygous for an increased expansion of an intronic GAA triplet repeat in the *FXN*gene [2], which represses frataxin expression via an epigenetic mechanism [3]. In these patients, most residual frataxin expression comes from the shorter GAA repeat expansion (GAA1), whose length explains 30–50% of the variability in age of symptoms onset and is a determinant of disease severity [4, 5]. FRDA is characterized by early atrophy of the posterior columns of the spinal cord, followed by progressive degeneration of the cerebellar dentate nuclei and their efferent fibers in the superior cerebellar pedunculi [6]. Clinically, affected individuals become overtly symptomatic only when cerebellar signs appear. Then, variable alterations in cerebellar, pyramidal, visual, auditory and cognitive systems contribute over time to the progression of neurological impaiment [7–9]. Cognition in FRDA is less studied than motor, sensory and gait disorders and awareness of potential cognitive impairment is low in both individuals with FRDA and caregivers. Recent investigations unveiled a prominent role of the cerebellum and its efferent tracts, emerging from the cerebellum dentate nuclei (DN), in perception, higher cortical functions and affect modulation [10, 11]. Thus, FRDA cerebellar pathology could be associated with cognitive impairment. Seminal investigations disclosed lower, but within normal limits, intellectual properties in individuals with FRDA compared to controls [12–14] and contemporary studies disclosed normal mini-mental state examination (MMSE) [15–20] or slightly abnormal MOntreal Cognitive Assessment (MOCA) [21, 22] scores leading to the belief that cognitive disorders in FRDA are relatively subtle and may not cause obvious functional impairment [7].

However, even if missed by classic screening tools, cognitive and affective impairments in FRDA may influence the ability of individuals with FRDA to study, work and develop intellectually and socially. This is why it is important to characterize the cognitive behavioural profile associated with FRDA to ensure that people with FRDA are able to maximize their potential in terms of cognitive function. Determining the extent and the longitudinal evolution of cognitive disorders in FRDA is in that context of paramount importance. The aim of this review and meta-analysis was to review (i) the studies that evaluated cognition domains in FRDA, (ii) explore possible relationships between clinical, genetic and imaging (structural and functional) characteristics and neuropsychological assessment, as well as (iii) the longitudinal evolution of cognitive change in FRDA.

## Methods

### Study selection

PRISMA guidelines were used in conducting this meta-analysis [23]. A literature search was performed using the databases Pubmed and Scopus to identify the relevant studies (January 1950–February 2021) using the combination of keywords as follows: Friedreich ataxia and cognition; Friedreich ataxia and attention; Friedreich ataxia and executive; Friedreich ataxia and language; Friedreich ataxia and memory; Friedreich ataxia and spatial; Friedreich ataxia and emotions; Friedreich ataxia and neuropsychologic. Reference lists of published reports were also reviewed for additional studies.

Inclusion criteria were genetically confirmed FRDA, controlled studies that used standardized neuropsychological tests and reported data from both individuals with FRDA and control participants as well as statistical tests results for outcomes measures (Mean, standard deviation (SD), number of subjects (n)).

### Statistical analysis

Mean and SD from studies using identical outcomes identified in at least two studies were pooled for the purpose of analysis [24, 25]. In addition, pooled effect sizes were calculated separately for neuropsychological tests reported in at least two studies with Cohen’s *d *[26, 27]. A random-effects model was chosen to obtain an average weighted effect size across the studies for each pooled domain. Pooled *d*-value, weighted for the sample sizes of the individual studies, was calculated for neuropsychological outcomes with a 95% confidence interval [26, 27]. Effect sizes were interpreted as small, medium and large for values of 0.2, 0.5 and 0.8 respectively based on convention [28]. Effect sizes were considered significant when the confidence interval (CI) did not contain zero.

### Data sharing agreement

Data can be shared upon reasonable request

## Results (Fig.1)

### Neuro-psychological evaluation (Table 1 summarizes the studies and the outcome measures included in the quantitative meta-analysis)

**Table 1:** Summary of the studies included in the quantitative meta-analysis in alphabetical order. In Bold, tests that were significatively different between FRDA patients and healthy controls. MMSE: mini-mental state examination; MOCA: MOntreal Cognitive Assessment; SMDT: Symbol Digit Modality Test; HSCT: Hayling sentence completion task; TMT: trail making test; TMT^R^: Reitan version of the TMT [41]; Stroop^G^: Golden version of the Stroop test [44]; DS: digital span; DSF: DS forward; DSB: DS backward; WAIS: Wechsler adult intelligence scale. III third version, R revised; RCPM Raven Colored Progressive Matrice. SPART 10/36 Spatial recall test. RAVLT, Rey Auditory Verbal Learning Test. SLD segment length discrimination task. Hayling sentence completion test HSCT. California verbal learning test, CVLT. PASAT, Paced Auditory Serial Addition Test. Wisconsin card sorting test WCST; ^C^: test corrected by patients’ PATA rate test and nine hole pegboard test score using the methods described in Sacca et al. [35]

	Patients/Controls	General	Verbal fluencies	Language	Attention/Executive	Attention and working memory	Memory and learning	Visuo-spatial	Emotion
Akhlagi [45]	12/14				**Simon Task**				
Cocozza [33]	24/24	**MOCA** **SDMT** ^**C**^	**Phonemic** ^**C**^ **Semantic** ^**C**^ **Naming nouns** **Pointing names**		**TMT** ^**C,R**^ **Stroop** ^**G**^ Attentive matrices test	DS	**SPART** **RAVLT**	RCPM **SLD** **Mental rotation**	
Cocozza [36]	19/20	**SDMT** ^**C**^			**TMT** ^**C,R**^		**SPART**	**SLD**	
Corben [20]	15/15	MMSE			TMT^R^ Stroop^G^				
Corben [42]	10/10				**TMT** ^**R**^ Stroop^G^				
Corben [43]	13/14				**Simon** **TMT** ^**R**^ **Stroop** ^**G**^				
Corben [39]	43/42			**HSCT**	**TMT** ^**R**^ Stroop^G^				
Costabile [22]	20/20	**MOCA**	**Phonemic** ^**C**^ **Semantic** ^**C**^		**Stroop test time interference index** **TMT** ^**R,C**^	DSF^WAIS-III^	**SPART** **RAVLT**		**Genova emotion recognition test**
De nobrega [16]	20/20	MMSE	**Phonemic** Semantic **Action verbal**						
Dogan [34]	22/22	MOCA	**Phonemic** ^**120**^ **Semantic** ^**120**^	Multiple choice vocabulary test	Stroop^G^	DSF/DSB **PASAT**	**CVLT**		**Faux pas test**
Georgiou [66]	13/14				**Simon**				
Klopper [46]	10/10				**Every day attention test**				
Mantovan [37]	13/13	IQ^**WAIS-R**^	**Verbal fluencies**	**Boston naming test**	**TMT** ^**R**^ **Stroop** **Attentive matrices test** **Tower of London**	**DSF/DSB** ^**WAIS-R**^			
Nachbauer [14]	29/28	Verbal IQ	**Phonemic** **Semantic**		**Tower of London** **Stroop**	DSF/DSB^WAIS-R^	Verbal learning and retention memory test	Incomplete letters Position discrimination	
Nieto [18]	26/31	MMSE	**Phonemic** **Semantic** **Action verbal**		**WCST**	**DSF**/DSB^WAIS-III^	**CVLT** **SPART** **Logical memory test**	Judgement line orientation test **Block design**	**Facial recognition test**
Sacca [35]	24/61	**SDMT**	**Phonemic** **Semantic**		**Attentional Matrices** **TMT** ^**R**^				
Shishegar [40]	21/28			**HSCT**	**TMT** ^**C,R**^ **Stroop** ^**G**^	**DSF/DSB** ^**WAIS-III**^			
Vavla [67]	21/18	**IQ** ^**WAIS**^							

On meta-analysis, eighteen studies evaluated, with standardized tests, the different cognitive domains in individuals with genetically confirmed FRDA compared to control participants devoid of neurological or psychiatric diseases. These studies, summarized in Table 1were included in the quantitative meta-analysis. Six studies were included for qualitative review despite the lack of controls due to large sample size of FRDA patients [29, 30], longitudinal follow-up [15, 19], practical composite outcome measure test [31] or structural correlation [32].

While standardized, the neuro-psychological test battery varied across the different studies but allowed data pooling, from at least two studies, for most outcome measures. Significant differences were found in all cognitive domains for individuals with FRDA compared to control participants. (Table 2, summarizes the pooled analysis)

**Table 2:** Pooled results of the studies included in the quantitative meta-analysis. MMSE: mini-mental state examination; MOCA: MOntreal Cognitive Assessment; TMT: trail making test; DSF: digital span forward; DSB: digital span backward; WAIS Wechler adult intelligence scale, III: third version, R: revised; CVLT: California verbal learning test; SPART: 10/36 Spatial recall test; RAVLT, Rey Auditory Verbal Learning Test; SLD: segment length discrimination task; *: Test corrected by patients’ PATA rate test and nine hole pegboard test score using the methods described in Sacca et al. [35] *d* : Cohen’s *d;* CI: confidence interval

Neuropsychological tests	Pooled test results	*p*	Pooled effect size (*d*,CI)	Number of studies	Number of patients
**SCREENING**					
MMSE	28,6±1,4 vs 29,3±0,98	0.0005	1.4 (1-1.8)	3	71
MOCA	23.9±3.4 vs 26.9±2	<0.0001	1.2 (0.8/1.5)	3	66
**VERBAL FLUENCIES**					
Phonemic Fluency	10,3±4,1 vs 14,4±4,3	<0.0001	1 (0.6-1.4)	2	49
Semantic verbal fluency	19,6±5,3 vs 24,2±4,8	<0.0001	1.13(0.8-1.4)	4	99
Semantic fluency, corrected^*^	18,9±5,8 vs 23,7±5	0.0001	1(0.6-1.4)	2	44
F-A-S	26,7±9,8 vs 41,9±5,6	<0.0001	2.2 (1.7-2.6)	2	60
F-A-S, corrected^*^	28,2±11,5 vs 41,5±9,1	<0.0001	1.3 (0.8-1.7)	2	44
Action verbal fluency	12,8±5 vs 18,8±5,8	<0.0001	1.3 (0.9-1.7)	2	56
**LANGAGE**					
Hayling sentence completion task	5,9±1,1 vs 6,4±0,7	0.00026	1.4 (1.1-1.8)	2	64
**ATTENTION/EXECUTIVE**					
Stroop interference score	54±9,4 vs 50,2 6,9	0.0003	0.5(0.3/0.7)	6	130
TMT (B-A)	44,4±30,8 vs 26,4±15	<0.0001	0.8 (0.4-1.1)	3	68
TMT A	137±143 vs 31,5±12	<0.0001	1.3 (0.8-1.7)	2	37
TMT B	213 ±162 vs 78 ± 31	<0.0001	1.4 (1-1.8)	2	37
Simon incongruent reaction time	861±219 vs 585 ± 131	<0.0001	1.6 (0.9-2.2)	2	25
TMT(B-A), corrected^*^	56,2±19,6 vs 33,9±10,6	<0.0001	1.4 (0.9-1.9)	2	41
Attentive Matrice test	44,9±10,2 vs 55,4±4,1	<0.0001	1.4(0.9-2)	2	37
**ATTENTION AND WORKING MEMORY**					
DSF^WAIS-III^	7,5±2,6 vs 8,3±0,9	0.0067	0.7 (0.4-1)	3	89
DSB^WAIS-III^	5,3 ±0,9 vs 6,4 ±1	<0.0001	1.4 (1-1.8)	2	59
DSF^WAIS-R^	6,9±1,3 vs 7,6±1,3	0.015	1(0.5-1.5)	2	42
DSB^WAIS-R^	5,5±1,2 vs 5,5±1,6	1	0 (-0.4-0.4)	2	42
**MEMORY AND LEARNING**					
CVLT	12,4±0,9 vs 12,9±0,6	0.2	0	2	48
SPART	18,9±6,2 vs 23,2 ± 3,5	<0.0001	1 (0.7-1.4)	3	64
**VISUO-SPATIAL**					
RAVLT	41,6±12,9 vs 47,6±9,3	0.014	0.5(0.2-0.9)	2	44
SLD	26,8±2 vs 28,7±2 1,6	<0.0001	1.3(0.8-1.7)	2	44

#### Cognitive screening

Cognitive screening was realized in thirteen out of the eighteen studies. Three studies used the MOCA [22, 33, 34], three studies used the MMSE [16, 18, 20], two studies used the Symbol Digit Modality Test (SDMT) [35, 36], two studies evaluated IQ [37, 38] and one study assessed verbal IQ [14]. Only results from MOCA and MMSE could be pooled as one study corrected SDMT score for ataxic and dysarthric impairments using patients’ PATA rate test and nine hole pegboard test score [36] and IQ scores were not evaluated using the same method in the three studies. On pooled analysis, FRDA patients displayed lower MOCA and MMSE scores with large effect size (23.9±3.4 vs 26.9±2, *p*<0.0001, *d*: 1.2 and 28.6±1.4 vs 29.3±0.98, *p*=0.0005, *d*: 1.4 respectively).

#### Verbal fluencies

Seven studies assessed phonemic and semantic fluencies [14, 16, 18, 22, 33–35], of which two [22, 33] corrected the results using patients’ PATA rate test and nine hole pegboard test score using the methods described in Sacca et al. [35] The raw values from Sacca et al, [35] were pooled with the uncorrected studies. Two studies assessed action verbal fluencies [16, 18] and one used a distinct verbal fluency from the aforementioned studies [37]. Verbal fluencies in all modalities were significatively poorer in FRDA patients compared to healthy controls with large effect size that were only marginally reduced by correction by PATA rate and nine hole pegboard test scores.

#### Language

Language was evaluated in four studies. The Hayling sentence completion task was used in two studies [39, 40], the Boston naming test in one [37] and a Multiple choice vocabulary test task in another [34]. Only in the Multiple choice vocabulary test task did individuals with FRDA performed similarly to controls [34].

#### Attention/executive

Sixteen studies assessed attention and/or executive functions. Eight studies used the Reitan’s [41] Trail making Test (TMT) [20, 22, 33, 36, 39, 40, 42, 43], with patients’ PATA rate test and nine hole pegboard test scores correction in three 22,33,36]. Six studies used the method developed by Golden [44] to calculate the Stroop interference score [20, 33, 34, 39, 40, 43], three studies used distinct and different declination of the Stroop test [14, 22, 37]. Simon’s task incongruent reaction time was reported in two studies [43, 45]. Attentive matrices test was used in two studies [33, 37], as was the Tower of London test [14, 37]. Wisconsin card sorting [18] and every day attention tests [46] were evaluated in two distinct studies. Pooled analysis disclosed lower performances in the TMT associated to a large effect size that grew larger after correction for ataxic symptoms. Individuals with FRDA showed pooled impaired performance on the Stroop interference score with a medium effect size.

#### Attention and working memory

Seven studies relied on the digital span to assess attention and working memory. Three studies used the digital span task included in the Wechsler adult intelligence scale, third version (WAIS-III) that starts with a sequence of two digits and ends with a sequence of nine digits [18, 22, 40], two studies used the task included in the revised Wechsler adult intelligence scale (WAIS-R) that starts with a sequence of three digits and ends with a sequence of eight digits [14, 37], and two did not specify the version used [33, 34]. One study used the Paced Auditory Serial Addition Task (PASAT) [34]. Pooled analysis disclosed significant differences in digital span forward (DSF) in both WAIS versions and backward (DSB) only in WAIS-III version. There, individuals with FRDA displayed lower performance compared to controls, associated to a medium (DSF) or high effect size (DSB).

#### Memory and learning

Six studies assessed memory and learning skills. The evaluation included the 10/36 Spatial recall test (SPART) in four studies [18, 22, 33, 36], the California verbal learning test (CVLT) in two [18, 34], the Verbal learning and retention memory test in one [14] and the logical memory test in one study [18]. Only SPART performances were lower in individuals with FRDA on pooled analysis but with a large effect size.

#### Visuospatial

Four studies included visuospatial skill tests. Those tests consisted in the Rey Auditory Verbal Learning Test (RAVLT) in two studies [22, 33], segment length discrimination (SLD) in two studies [33, 36], Raven Colored Progressive Matrice and mental rotation in one study [33], Incomplete letters and position discrimination tests in one [14], and Judgement line orientation, Facial recognition test and Block design in one study [18]. FRDA patients performed worse than controls with large size effect for the SLD and medium size effect for the RAVLT.

#### Emotion recognition and social cognitive abilities

Emotion recognition and social cognitive abilities were evaluated in three studies using different outcome measures including the Social Cognitive and Emotional Assessment and Ekman facial expression recognition Test [30]; the Faux-pas test (*n*=22) [34] and the Geneva Emotion Recognition Test (*n*=20) [47]. Individuals with FRDA compared to control participants, were less efficient in the Faux-pas test (*n*=22) [34] and Geneva emotion recognition test (*n*=20) [47].

#### Correlations between cognitive function and clinical parameters

Data from thirteen studies were identified as appropriate to include in potential correlations between neuropsychological test results and clinical parameters such as GAA1 (allele with smaller GAA repeat size), age of symptom onset (ASO), disease duration (DD) and clinical scales (Friedreich Ataxia Rating Scale (FARS), Scale for the Assessment and Rating of Ataxia (SARA)).

Ciancarelli et al. found no correlation between memory and phonemic verbal fluency test results and disease duration (*n*=24) [19]. Corben et al. reported a significant negative correlation between ASO and the incongruency effect in a Simon task in two consecutive studies from their group (*n*=13; *n*= 12) but no other significant correlations with GAA1, DD and the FARS score [43, 48]. In addition a later work did not identify a correlation between ASO, DD, GAA1 or the FARS score with TMT and Stroop inferences scores (*n*=43) [39]. Dogan et al. found a significant correlation between impaired phonemic fluency performance and longer DD but no correlation between tests of memory, attention, executive and social cognition and clinical parameters (*n*=22) [21]. Similarly in a large European cohort (*n*=592), a correlation between DD and phonemic fluency was identified [49]. Klopper et al. disclosed significant correlations between subtests of every day attention and GAA1 and the FARS score but not with DD nor ASO (*n*=16) [46]. Mantovan et al. described poorer Stroop and Tower of London performances in individuals with longer DD, however did not find similar correlations with memory, language and calculation tests. Moreover, GAA1 was not correlated to any neuropsychological measure while clinical scores like the FARS or SARA were not reported (*n*=13) [37]. Sayah et al., found correlations between measures of attention and the SARA, DD and GAA1 (*n*=46) [30].

Two longitudinal studies looked at the evolution of neuropsychological test results over time. Shishegar et al. found worsening performances for TMT (B-A) in individuals with FRDA over 24 months, but no degradation in working memory or executive function. In addition no correlation between clinical parameters and neuropsychological test results were identified (*n*=21) [40]. In the EFACTS cohort, over a two-year follow-up, no significant decline was found in verbal fluency [50]. Yet, with longer follow-up, in a study where individuals with FRDA were assessed on average with an eight year separation, Hernandez-Torres et al. (*n*=39) found worse performances for the Stroop interference task, phonemic fluency tests and processing speed, while memory or visuospatial skills remained stable (*n*=29) [15].

Finally, two studies correlated composite scores based on combined neuropsychological test results. Nachbauer et al. found correlation with an executive score that included attention, executive, verbal fluency and visuospatial items and GAA1, ASO and the SARA score (*n*=29) [14]. Naeije et al. described a tight correlation between the cerebellar cognitive affective syndrome (CCAS) scale score and the SARA score (*n*=19) [31].

#### Correlations between cognitive performances and structural parameters

Six studies sought correlations between imaging measures and cognitive performances.

Ahklagi et al. (*n*=12), in a magnetic resonance imaging (MRI) and tractography study exploring reaction time and Simon (incongruence) effect in individuals with FRDA and controls found that, the mean and radial diffusivity of the dentato-rubral tract was positively correlated with choice reaction time, congruent reaction time, incongruent reaction time and Simon effect reaction time and negatively with the larger GAA1 [48]. Cocozza et al. (*n*=19), in a voxel‐based morphometry and volumetric MRI study, found a direct correlation between cerebellar Lobule IX volume and impaired visuo-spatial functions but no correlations between structural parameters and executive and memory test results [36]. A former study by the same authors found no significant correlation between functional MRI (fMRI) resting state connectivity (rsFC) study (*n*=24) and language, memory, executive and visuospatial tests [33].

Dogan et al. (*n*=22), in a fMRI and diffusion tensor imaging study combining language, memory, attention, executive and social cognition tests only showed in, post hoc correlations, a significant negative association between right cerebellar Crus I and left Brodmann area 44 and left insula functional activities for phonemic fluency execution in individuals with FRDA [34]. Harding et al. (*n*=29), using a verbal n-back working memory task in fMRI, disclosed that task-related activation in the right dentate nucleus was significantly associated with a composite clinical index score based on ASO, the FARS score, DD and GAA1. This effect was most pronounced with respect to the FARS score and GAA1 [32]. In the longitudinal assessment of the same cohort, Shishegar et al. (*n*=21), found no significant correlations between longitudinal change in neurocognitive measures and change in brain activation over time [40].

## Discussion

This meta-analysis confirmed that individuals with FRDA show significantly lower performances than control participants in the cognitive domains of language, attention, executive, memory, visuospatial perception, emotion recognition and social cognitive abilities.

Despite these results, awareness of potential cognitive disorders in individuals with FRDA appears low. Several reasons may explain why cognitive disorders in FRDA are so often overlooked. A possible interpretation relates to the fact that cognitive disorders are considered relatively subtle and do not cause obvious functional impairment [7]. Indeed, the deficits described do not generally preclude a person with FRDA from participating in education at school and college/university, gaining meaningful and at times cognitively demanding employment, partnering and raising a family [51]. Another explanation might be the *a priori* fear of affective, social and professional negative repercussions that may develop with increased awareness of cognitive disorders in FRDA. This is an even more sensitive issue if we are to consider that cognitive impairments may progress significantly along disease course which could prevent potential employers or partners to engage in the long run with individuals with FRDA. Yet, even if missed by classic screening tools, cognitive and affective impairments in FRDA may impact the ability to study, work and develop both intellectually and socially, in potentially large numbers of individuals with FRDA. Therefore, cognitive impairment should be considered when difficulties arise in any of those fields. Prompt recognition of cognitive difficulties related to FRDA pathology, in those circumstances, could provide an objective explanation to the problem encountered by the affected individual providing opportunity for dedicated follow-up and care.

The widespread impairment of cognitive function in individuals with FRDA suggests a cerebellar component in the pathophysiological mechanism of the cognitive impairments or in their modulation for several reasons. First, the combination of relatively mild but global higher neocortical dysfunction is characteristic of the Cerebellar Cognitive and Affective Syndrome (CCAS). The CCAS, first described over two decades ago, comprises a form of thought dysmetria that hampers language, emotional regulation, memory, attention, visuospatial and executive function [52, 53]. Interestingly, a CCAS screening and follow-up scale was designed in 2018 based on the neuropsychological tests that could most efficiently single out individuals with cerebellar pathology from healthy individuals [52]. The selected tests for that CCAS Scale bear a striking similarity to tests that proved to have the largest size effect in this meta-analysis, underlining a probable similar origin for CCAS and FRDA cognitive profile [52]. Verbal fluencies were among the tests that displayed the largest effect size in our meta-analysis paralleling the impairment of verbal fluency in the large uncontrolled EFACTS cohort [50]. Verbal fluency represent three of the ten items that constitute the CCAS scale as well as half the points of its raw score highlighting the difficulties that individuals with cerebellar disorders have with verbal fluencies. Whether verbal fluencies reflects mostly impaired language performances [52, 54], altered executive functions [55, 56] or both is still debated [57]. In cerebellar impairment either language and/or executive function could be responsible. The cerebellum is involved in language processing tasks [58, 59] and patients with acute or degenerative disorders involving posterior cerebellar lobes display various language deficits [60–62]. Similarly, executive functions are also known to be impaired in cerebellar disorders [63]. In our pooled analysis, the TMT and the Stroop interference score showed lower effect size than verbal fluencies tasks, reflecting the fact that significant impairments in FRDA patients were inconsistently found and probably less salient than language dysfunction. Thus, in FRDA, verbal fluencies alterations probably reflects more language than executive dysfunctions. Yet future studies, using purer language tasks like the Peabody Picture Vocabulary Test, that assess receptive vocabulary, could help disentangle the respective contribution of executive and language roles in FRDA impaired verbal fluencies [64].

The neuropathology of FRDA , is hallmarked by progressive DN atrophy with relative sparing of cerebral hemispheres [6]. The cerebellum through cerebello-cortical loops is known to regulate most components of cognition, and impairments of cerebellar posterior lobes or dentato-thalamic pathways are associated with the CCAS [60, 63]. Thus, DN pathology in FRDA is a likely candidate in the genesis of cognitive impairment in FRDA. Correlations between neuropsychological impairments in FRDA and central nervous system structures alterations, where significant despite limited sampling size, and were specifically related to the DN, dentato-rubral tracts and posterior cerebellar lobes [58, 60]. Finally, exploration of correlations between clinical parameters and cognitive performances confirmed that the increment in cognitive impairment over time paralleled ataxia severity. Taken together these findings suggest that progressive DN atrophy and efferent tracts impairment in FRDA are probably responsible for a progressive cognitive pattern pertaining to the CCAS, whose severity parallels rating on cerebellar ataxia severity scales. Yet, direct evidence for a cerebellar role in FRDA cognitive impairments are still missing. A potential way to confirm this hypothesis could be through resting state connectivity (rsFC) in FRDA patients. Indeed, ctDCS has been found recently to improve cognitive performances in a population of patients (one FRDA) with cerebellar ataxia of mixed origins [65], supporting the role of the cerebellum in their modulation.

This analysis provides, only limited evidence on potential progression of cognitive impairments in individuals with FRDA and the relationship to cerebral and cerebellar structure and function. Dedicated longitudinal studies are crucially needed in order to better understand the cognitive profile of FRDA. Further research in this area, using either the CCAS‐scale [31, 52] or a combination of the tests with the highest effect size as highlighted in this meta-analysis, could provide an opportunity to confirm previous findings, particularly regarding the relationship between structural/functional imaging and cognitive impairment as well as the onset and longitudinal evolution of cognitive symptoms. This would allow better evaluation of cognitive disorder in FRDA and help design appropriate interventions to mitigate the impact of cognitive impairment on functional capacity. Indeed, understanding the neurobehavioral profile associated with FRDA is fundamental for intervention aimed at improving independence, school, academic and vocational capacity and thus quality of life.

## Conclusions

Individuals with FRDA display significantly lower performances in many cognitive domains compared to control participants. The spectrum of the cognitive profile alterations in FRDA and its correlation with disease severity and cerebellar structural parameters suggest a cerebellar role in the pathophysiology of FRDA cognitive impairments.

**Fig.1:**
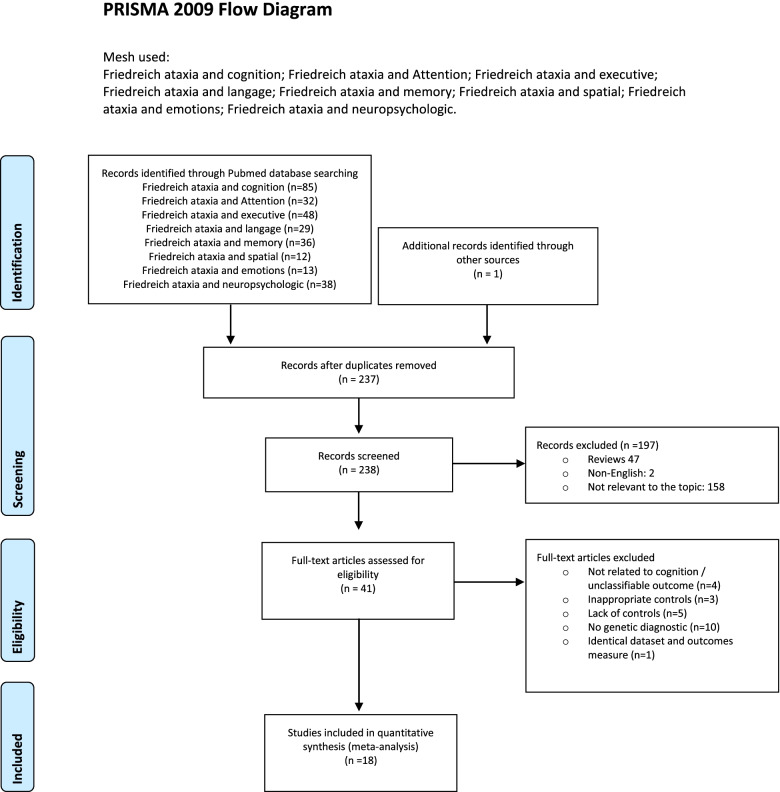
PRISMA 2009 Flow Diagram. Mesh used: Friedreich ataxia and cognition; Friedreich ataxia and Attention; Friedreich ataxia and executive; Friedreich ataxia and langage; Friedreich ataxia and memory; Friedreich ataxia and spatial; Friedreich ataxia and emotions; Friedreich ataxia and neuropsychologic

## Data Availability

The datasets used and/or analysed during the current study available from the corresponding author on reasonable request.

## Abbreviations

SPART: 10/36 Spatial recall test
ASO: Age of symptom onset
CVLT: California verbal learning test
CCAS: Cerebellar cognitive affective syndrome
CI: Confidence interval
DN: Dentate nuclei
DSB: Digital span backward
DSF: Digital span forward
DD: Disease duration
FRDA: Friedreich ataxia
FARS: Friedreich Ataxia Rating Scale
GAA1: GAA repeat expansion
MMSE: Mini-mental state examination
MOCA: Montreal Cognitive Assessment
PASAT: Paced Auditory Serial Addition Task
rsfc: Resting state connectivity
SARA: Scale for the Assessment and Rating of Ataxia
SLD: Segment length discrimination
SDMT: Symbol Digit Modality Test
TMT: Trail making Test
WAIS-R: Wechsler adult intelligence scale
